# Delayed Diagnosis of Tuberous Sclerosis Complex Presenting as Abnormal Uterine Bleeding: A Case Report and Literature Review

**DOI:** 10.1002/ccr3.73438

**Published:** 2026-09-03

**Authors:** Kidus Tesfaye Bezabih, Hana Sisay Belay, Merid Lemma Kebede, Abebe Ketema Aderu, Mathyas Dessalegn Gebreyes, Kelil Hussein Jibicho, Adugna Assefa Talemahu, Elias Fikre Merid, Kedir Negesso Tukeni

**Affiliations:** ^1^ Department of Internal Medicine Jimma University Jimma Ethiopia; ^2^ Department of Pediatrics and Child Health Jimma University Jimma Ethiopia; ^3^ Department of Radiology Jimma University Jimma Ethiopia; ^4^ Department of Urology Jimma University Jimma Ethiopia; ^5^ Department of Gynecology and Obstetrics Jimma University Jimma Ethiopia

**Keywords:** abnormal uterine bleeding, angiomyolipoma, Ethiopia, lymphangioleiomyomatosis, PEComa, tuberous sclerosis complex

## Abstract

Tuberous sclerosis complex (TSC) is a rare multisystem genetic disorder characterized by the development of hamartomatous lesions in multiple organs. Although neurologic and dermatologic manifestations commonly lead to diagnosis during childhood, some patients remain undiagnosed until adulthood because of atypical presentations. We report the case of a 28‐year‐old Ethiopian woman with a longstanding seizure disorder who presented with a two‐year history of abnormal uterine bleeding. Physical examination revealed facial angiofibromas and hypomelanotic macules. Further evaluation demonstrated bilateral renal angiomyolipomas, pulmonary lymphangioleiomyomatosis, cortical and subcortical brain lesions, and uterine myometrial lesions compatible with perivascular epithelioid cell tumors (PEComas). Histopathologic examination of a surgically excised renal mass confirmed angiomyolipoma. Based on the presence of multiple major clinical and radiologic features, the patient fulfilled the 2021 International Tuberous Sclerosis Complex diagnostic criteria for a definite diagnosis. This case highlights abnormal uterine bleeding as an uncommon sentinel manifestation of TSC and emphasizes the importance of considering syndromic diagnoses when gynecologic abnormalities coexist with multisystem findings. Early recognition facilitates multidisciplinary management and long‐term organ‐specific surveillance.

## Key Clinical Message

Abnormal uterine bleeding may represent an uncommon presenting manifestation of tuberous sclerosis complex. When gynecologic pathology occurs alongside characteristic dermatologic, neurologic, renal, or pulmonary findings, clinicians should consider an underlying syndromic diagnosis. Early recognition of multisystem involvement enables timely diagnosis, multidisciplinary management, and appropriate long‐term surveillance.

**Abbreviations**
AML: Angiomyolipoma
ANA: Antinuclear antibody
CT: Computed tomography
dsDNA: Double‑stranded DNA
HRCT: High‑resolution computed tomography
LAM: Lymphangioleiomyomatosis
MRI: Magnetic resonance imaging
TSC: Tuberous sclerosis complex

## 1 Introduction

Tuberous sclerosis complex (TSC) is an autosomal dominant tumor‐predisposition syndrome caused by pathogenic variants in *TSC1* or *TSC2*, leading to dysregulated mammalian target of rapamycin (mTOR) signaling and the development of hamartomas and neoplasms across multiple organ systems [1, 2, 3]. Its clinical spectrum is broad, ranging from epilepsy and neurocognitive manifestations to characteristic dermatologic lesions and renal, pulmonary, cardiac, and ophthalmologic involvement [1, 2]. The diagnosis is often established during childhood when seizures, characteristic imaging findings, or cutaneous stigmata prompt further evaluation. However, some individuals remain undiagnosed until adulthood, particularly when neurologic manifestations are mild and extracranial features evolve gradually [2, 4, 5].

In women with TSC, gynecologic manifestations are uncommon but increasingly recognized. These include uterine perivascular epithelioid cell tumors (PEComas), hamartomatous uterine lesions, ovarian cysts, and menstrual irregularities [6, 7, 8, 9, 10]. Because these manifestations frequently mimic more common gynecologic conditions such as adenomyosis, leiomyomas, or dysfunctional uterine bleeding, TSC is rarely considered during the initial diagnostic evaluation [6, 7, 8, 9, 10]. Consequently, opportunities for syndrome‐based diagnosis, counseling, and organ‐specific surveillance may be missed.

Previous reports have described diverse presentations leading to the diagnosis of TSC, including seizures, dermatologic lesions, renal angiomyolipomas, and incidental radiologic findings. In contrast, abnormal uterine bleeding (AUB) as the primary presenting complaint remains rarely reported and may obscure recognition of the underlying multisystem disorder. The differential diagnosis of AUB in young women typically focuses on common gynecologic, endocrine, or hematologic causes, often delaying consideration of syndromic conditions such as TSC [4, 10, 11, 12, 13].

We report a 28‐year‐old Ethiopian woman who presented with a two‐year history of abnormal uterine bleeding and was subsequently found to have multisystem manifestations of TSC, including facial angiofibromas, hypomelanotic macules, renal angiomyolipomas, pulmonary lymphangioleiomyomatosis, and intracranial lesions. This case highlights abnormal uterine bleeding as an uncommon sentinel manifestation of TSC and underscores the importance of careful clinical examination and multidisciplinary evaluation in patients with atypical gynecologic presentations.

## 2 Case History and Examination

A 28‐year‐old nulliparous Ethiopian woman presented with a two‐year history of heavy menstrual bleeding associated with intermenstrual spotting, progressive fatigue, and lower abdominal pain. The bleeding had gradually worsened over time and was associated with symptoms suggestive of chronic blood loss. She denied a previous diagnosis of gynecologic disease and had no known family history of tuberous sclerosis complex (TSC) or other hereditary disorders.

Her past medical history was significant for a seizure disorder diagnosed at 12 years of age, for which she had been receiving phenobarbitone 100 mg daily with good seizure control. There was no history of chronic respiratory disease, renal disease, hypertension, diabetes mellitus, or previous surgical intervention. She had not undergone prior evaluation for a syndromic or genetic disorder despite her longstanding neurologic history.

On physical examination, the patient appeared chronically ill and pale. Vital signs were within normal limits. Dermatologic examination revealed multiple facial angiofibromas distributed over the malar regions and nose, along with several hypomelanotic macules on the trunk and extremities. Abdominal examination demonstrated mild suprapubic tenderness without signs of peritonitis. Pelvic examination revealed an enlarged and irregular uterus. No focal neurological deficits were identified, and cardiovascular and respiratory examinations were unremarkable.

The coexistence of abnormal uterine bleeding, a longstanding seizure disorder, and characteristic cutaneous lesions raised suspicion for an underlying multisystem disorder, prompting further diagnostic evaluation.

### 2.1 Differential Diagnosis

In a young woman presenting with persistent abnormal uterine bleeding (AUB), common gynecologic causes such as uterine leiomyomas, adenomyosis, endometrial hyperplasia, ovulatory dysfunction, and endometrial polyps were initially considered. These conditions account for the majority of cases of chronic AUB and are frequently prioritized during gynecologic evaluation.

Systemic causes of abnormal bleeding were also considered. Hematologic disorders, including coagulopathies and platelet dysfunction, as well as endocrine abnormalities such as thyroid disease, may contribute to abnormal menstrual bleeding patterns. Autoimmune disorders, particularly systemic lupus erythematosus, were entertained because of elevated antinuclear antibody (ANA) and double‐stranded DNA (dsDNA) titers. However, the absence of compatible clinical manifestations made an autoimmune etiology unlikely.

Malignant conditions, including endometrial carcinoma and uterine sarcoma, were considered because of the persistent nature of the bleeding and the presence of uterine abnormalities on imaging studies. Nevertheless, endometrial biopsy did not reveal evidence of malignancy.

As the evaluation progressed, the coexistence of facial angiofibromas, hypomelanotic macules, a longstanding seizure disorder, bilateral renal masses, pulmonary cystic lesions, and intracranial abnormalities suggested a multisystem disorder. The constellation of findings ultimately supported a diagnosis of tuberous sclerosis complex (TSC), with uterine perivascular epithelioid cell tumors (PEComas) representing the likely cause of the abnormal uterine bleeding.

### 2.2 Investigations

Initial laboratory evaluation demonstrated iron‐deficiency anemia with a hemoglobin level of 8.2 g/dL. Additional laboratory findings are summarized in Table 1.

**TABLE 1:** Laboratory inventions of the patient during the initial presentation and subsequent workups.

Variables	Results	Normal references
Hematocrit (%)	24.6	36.0–46.0
Hemoglobin (mg/dL)	8.2	11–16.5
White cell count (per μL)	5900	4000–15,000
Neutrophil, *n* (%)	4194 (71.1)	1800–7700
Lymphocyte, *n* (%)	1286 (21.8)	1000–4800
Monocytes, *n* (%)	1180 (0.2)	200–1200
Eosinophils, *n* (%)	0.0	0–900
Basophils, *n* (%)	0.0	0–300
Platelet (per μL)	439,000	150,000–450,000
Mean corpuscular volume (fL)	71	80–100
Mean Corpuscular Hemoglobin (pg)	23 pg	28–32
Mean Corpuscular Hemoglobin concentration (g/dL)	30	33–36
Red‐cell distribution width (%)	18	9–14.5
ESR (mm/h)	32	0–20
Creatinine (mg/dL)	1.0	0.5–1.2
Blood urea nitrogen (mg/dL)	22	7–20
Sodium (mmol/L)	135	135–145
Potassium (mmol/L)	3.5	3.5–5.5
Chloride (mmol/L)	100	98–107
Calcium (mg/dL)	8.56	8.5–10.5
AST (IU/L)	40	0–40
ALT (IU/L)	37	0–41
ALP (IU/L)	128	40–130
LDH (U/L)	250	140–280
Serum Bilirubin (mg/dL) Total	1.18	0.3–1.2
Serum Bilirubin (mg/dL) direct	0.12	0.0–0.2
Random blood sugar (mm/g/dL)	88	70–110
Prothrombin time (s)	10.1	10–14
APTT (s)	27	25–30
INR	0.84	0.7–1.2
TSH (m IU/mL)	2.9	0.3–4.5
FT3 (pg/mL)	2.96	2.0–4.2
FT4 (pg/mL)	1.15	0.9–1.95
Rheumatoid factor quantitative (U/mL)	< 20	< 20
ASO titer	Non‐reactive	Negative
ANA quantitative (AU/ml)	400	0–40
anti‐dsDNA (AU/mL)	120	0–20
HIV test	Non‐reactive	Non‐reactive
Vit‐B12 (pg/mL)	512	200–800
Serum folate (ng/mL)	18	1.8–9
Direct Coombs test	Negative	
Serum VDRL test	Non‐reactive	Non‐reactive
Hepatitis B surface antigen test	Negative	Negative
Hepatitis C antibody test	Negative	Negative
Urinalysis	Microscopic few RBCs + dipstick blood +2 Others are negative	
Stool H Pylori test	Negative	Negative
Serum Albumin (g/dL)	4.1	3.5–5.5

Although antinuclear antibody (ANA) and double‐stranded DNA (dsDNA) titers were elevated, the patient had no clinical features suggestive of systemic lupus erythematosus or other autoimmune disease. These serologic abnormalities were therefore considered nonspecific.

**FIGURE 1:**
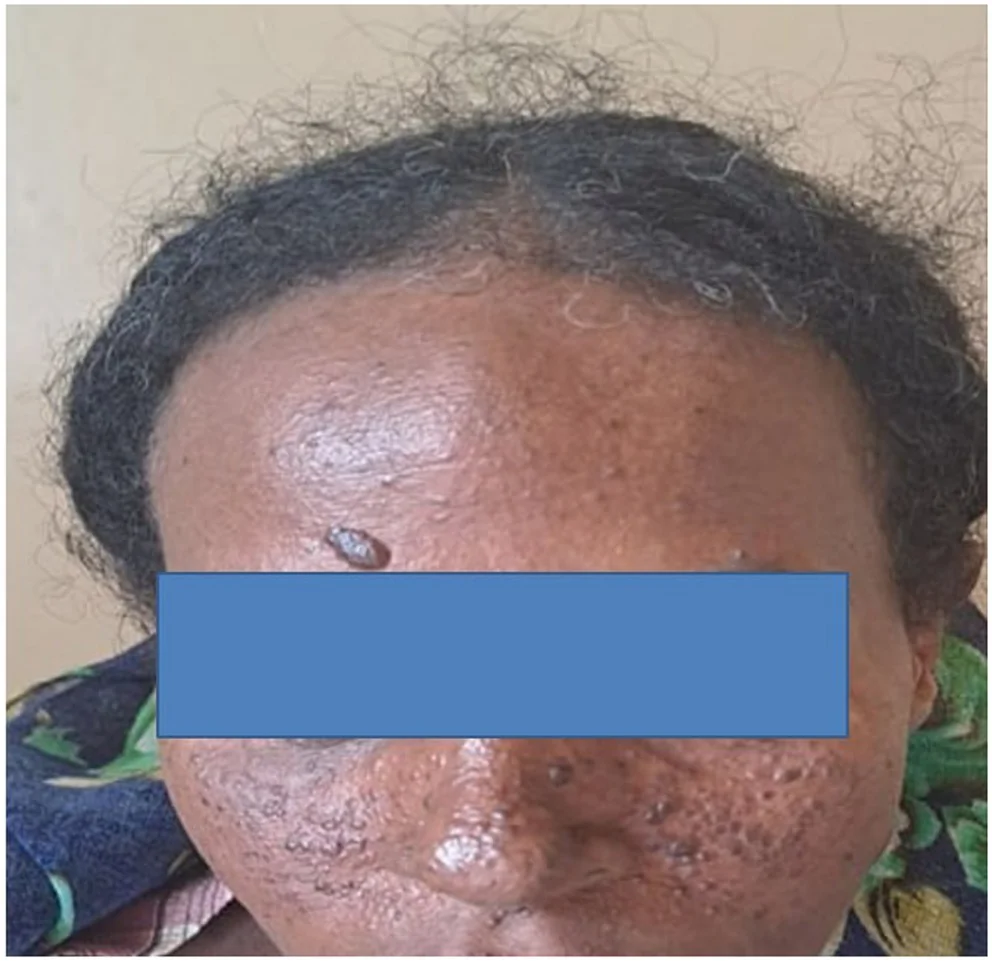
Facial angiofibromas and hypopigmented macules observed on physical examination. These cutaneous findings are characteristic of tuberous sclerosis complex (TSC).

As part of the gynecologic evaluation, transvaginal ultrasonography demonstrated uterine masses suggestive of hamartomatous lesions, a thickened endometrium containing intrauterine clots, associated ovarian cysts, and pelvic free fluid. Endometrial biopsy was subsequently performed and revealed no significant histopathologic abnormality. Physical examination identified multiple facial angiofibromas and hypomelanotic macules (Figure 1), prompting consideration of an underlying neurocutaneous syndrome.

**FIGURE 2:**
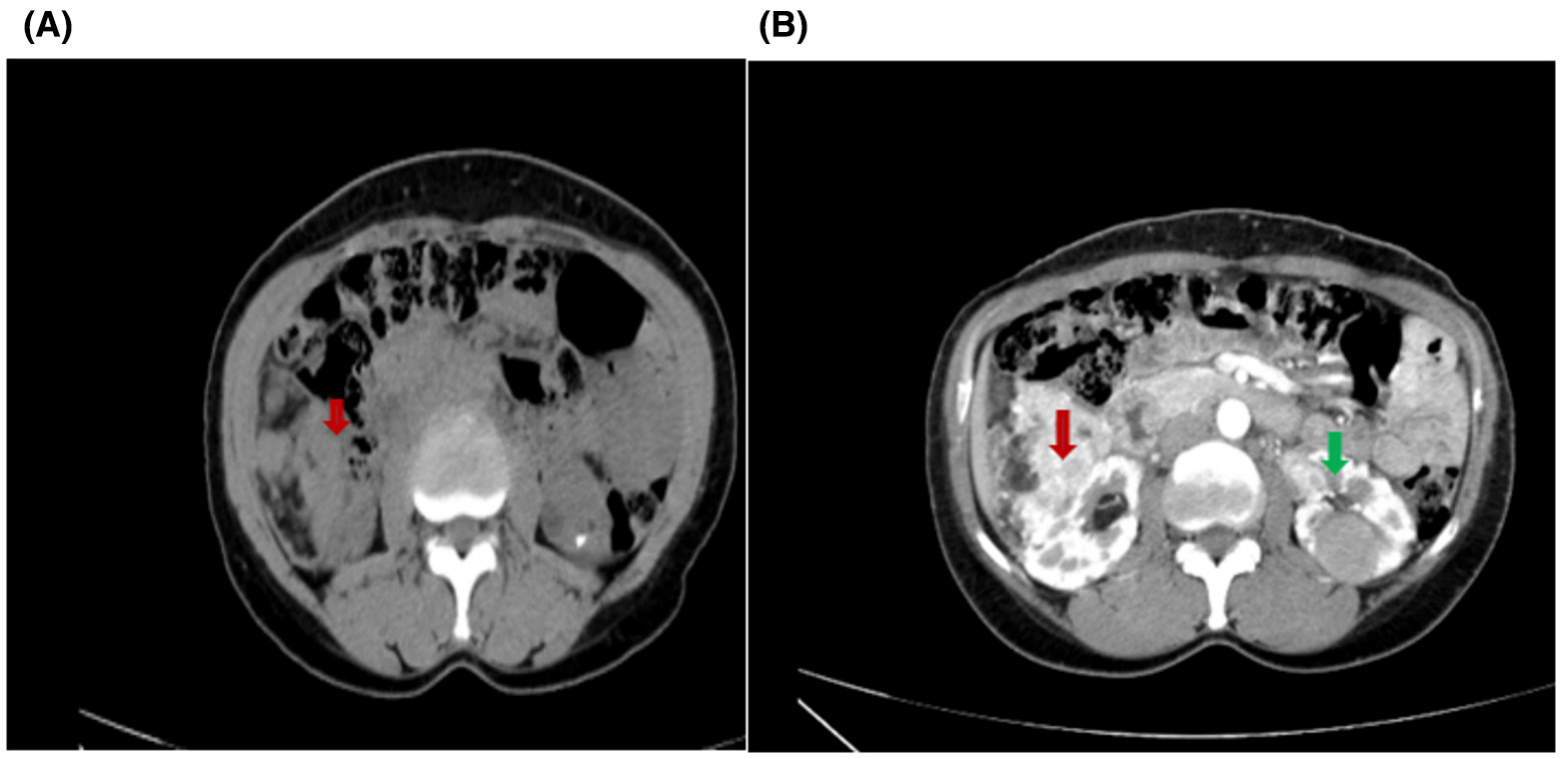
*Imaging findings of the case of TSC* (A) Pre contrast Abdominal CT. (B) Post contrast CT of the abdomen showing bilateral renal cortical angiomyolipomas (AML), identified as hypo dense, enhancing right renal cortical mass with internal fat (red arrow). Additionally, there are two hyper dense, enhancing left renal cortical masses (green arrow). AML = angiomyolipoma; LAM = lymphangioleiomyomatosis.

Abdominal ultrasonography demonstrated multiple echogenic and hypoechoic cortical renal lesions. Triphasic computed tomography (CT) of the abdomen further characterized these findings, revealing a 6 × 5.4 cm fat‐containing enhancing right renal cortical mass and two additional enhancing left renal cortical masses measuring 28 × 24 mm and 27 × 24 mm, respectively, consistent with bilateral renal angiomyolipomas (Figure 2).

**FIGURE 3:**
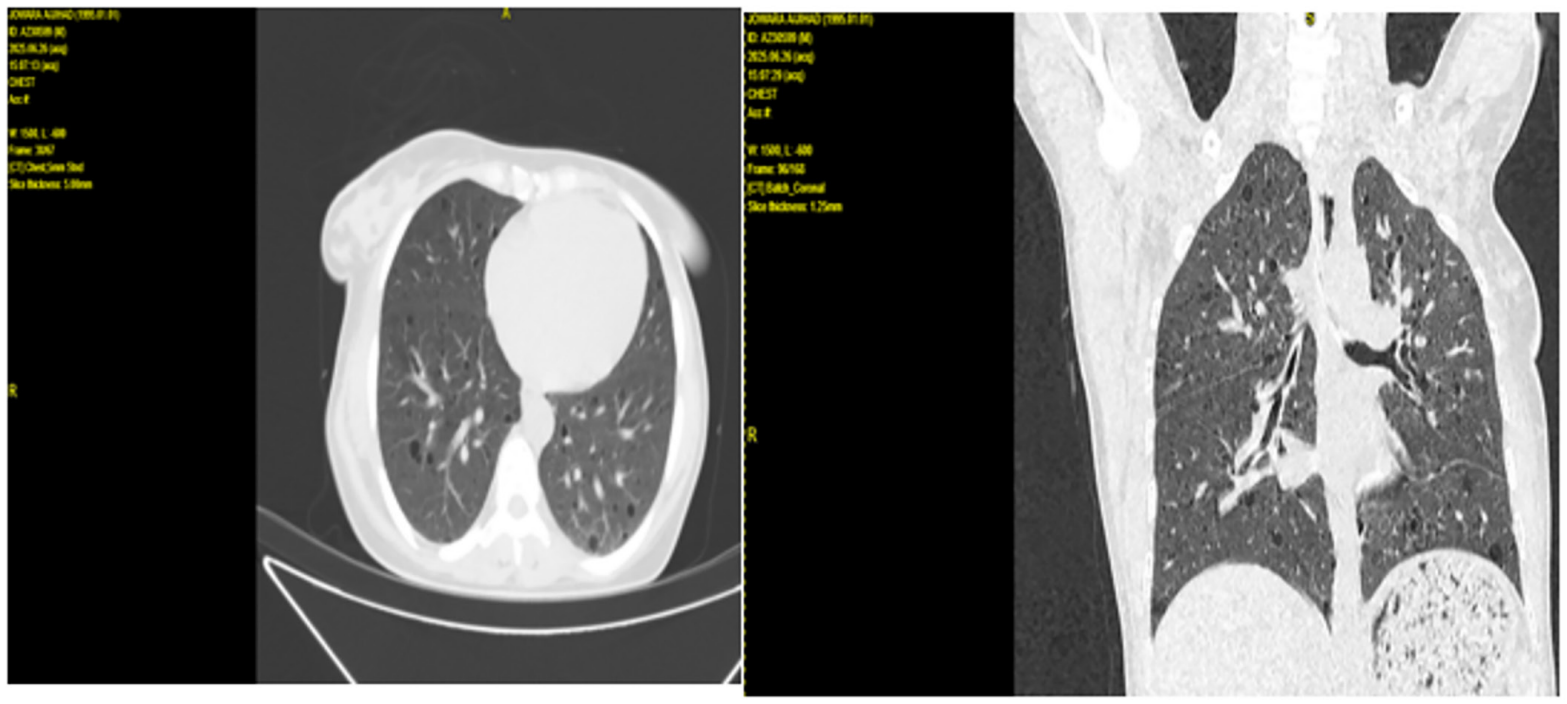
HRCT of the chest reveals bilateral thin‐walled, round lung cysts in the upper, middle, and lower lobes, indicated by red arrows. The conclusion suggests these findings are likely indicative of Lymphangioleiomyomatosis (LAM).

High‐resolution computed tomography (HRCT) of the chest demonstrated numerous bilateral thin‐walled cysts distributed throughout the upper, middle, and lower lung zones, findings consistent with lymphangioleiomyomatosis (LAM) (Figure 3).

**FIGURE 4:**
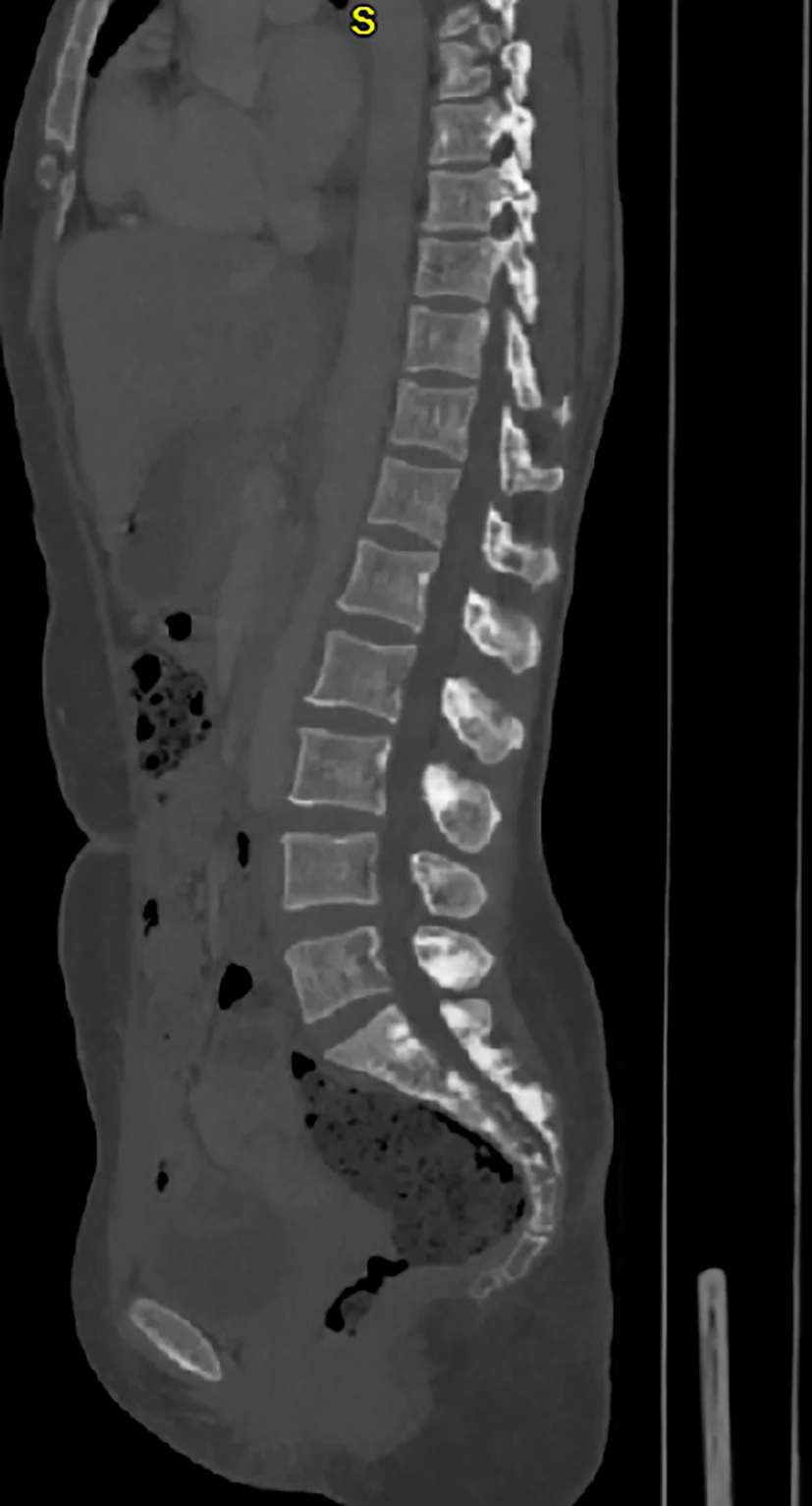
CT scan of the chest and spine showed that diffuse posterior element sclerosis is observed in the thoracic and lumbar spine.

A concurrent CT examination of the thoracic and lumbar spine demonstrated diffuse sclerosis involving the posterior spinal elements (Figure 4).

**FIGURE 5:**
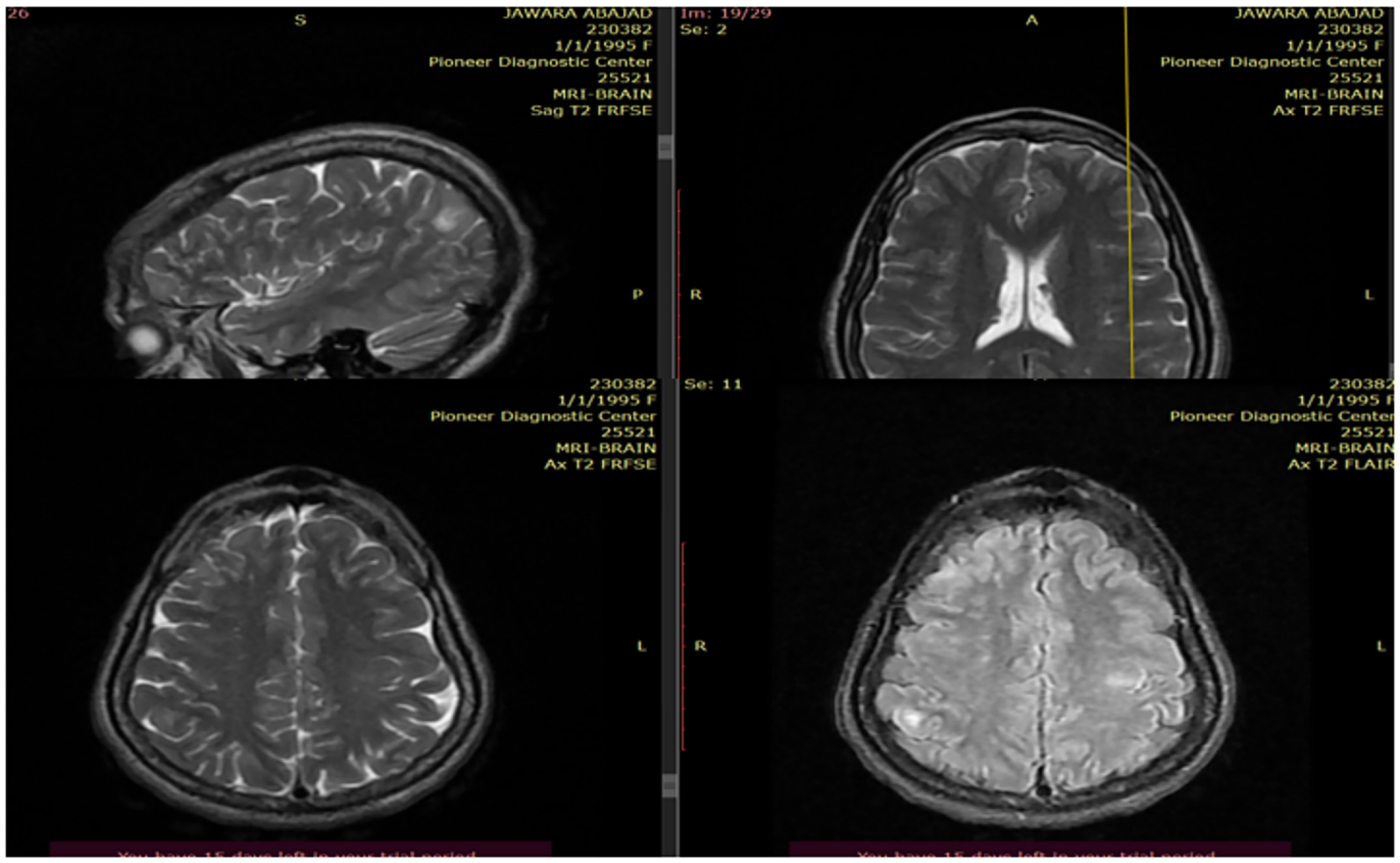
MRI of the brain: A small subependymal nodule is noted in the lateral ventricle with T1/T2 isointense signal. Multiple patchy abnormal signal changes are present in the cortical and subcortical regions of the bilateral frontal, parietal, and posterior temporal lobes, showing no contrast enhancement.

Given the multisystem findings, brain magnetic resonance imaging (MRI) was performed. Imaging demonstrated a small subependymal nodule within the lateral ventricle together with multiple cortical and subcortical lesions involving the bilateral frontal, parietal, and posterior temporal lobes, findings compatible with cortical tubers and subependymal involvement associated with TSC (Figure 5).

Further gynecologic assessment using pelvic MRI demonstrated multiple uterine myometrial lesions with imaging characteristics compatible with perivascular epithelioid cell tumors (PEComas), together with associated pelvic involvement.

**FIGURE 6:**
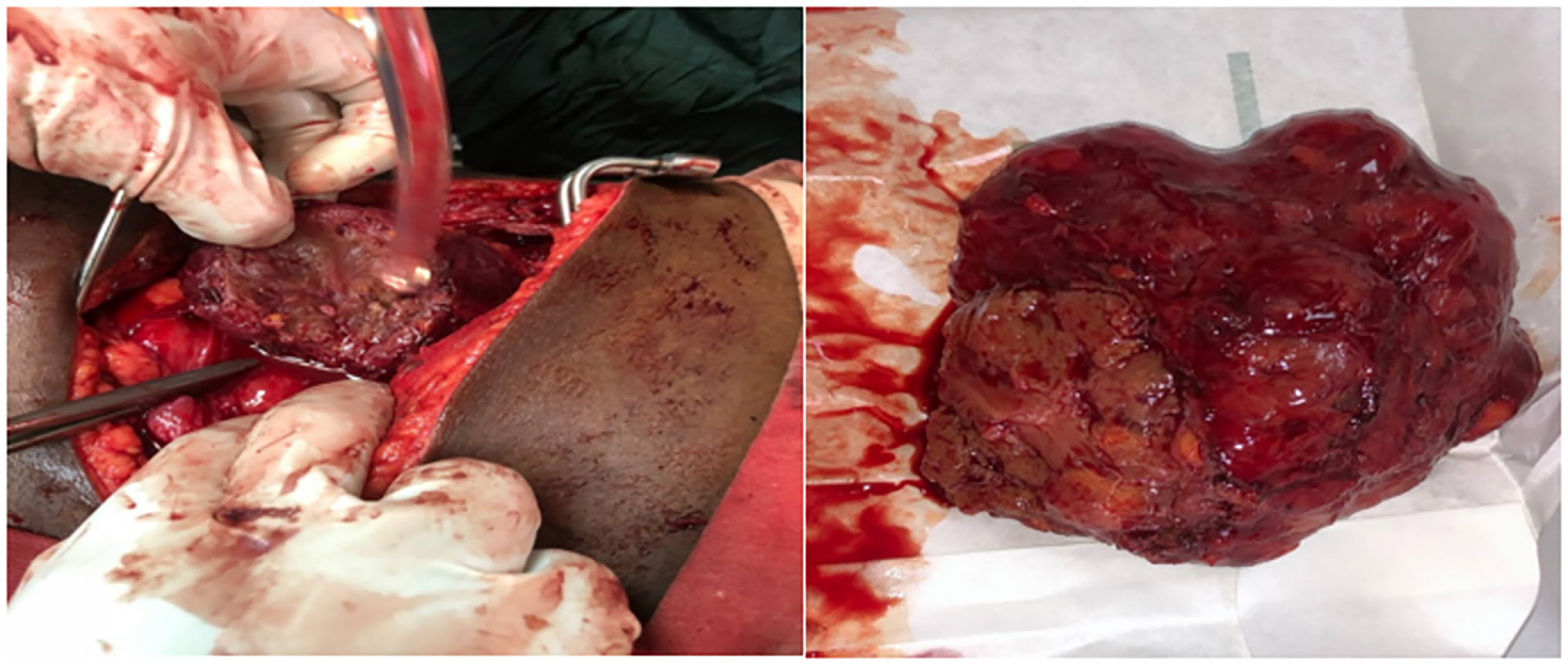
Surgery, specifically a partial nephrectomy, was performed to address the patient's worsening bilateral flank pain. A mass measuring 8 cm × 7 cm × 5 cm was removed from the left kidney.

**FIGURE 7:**
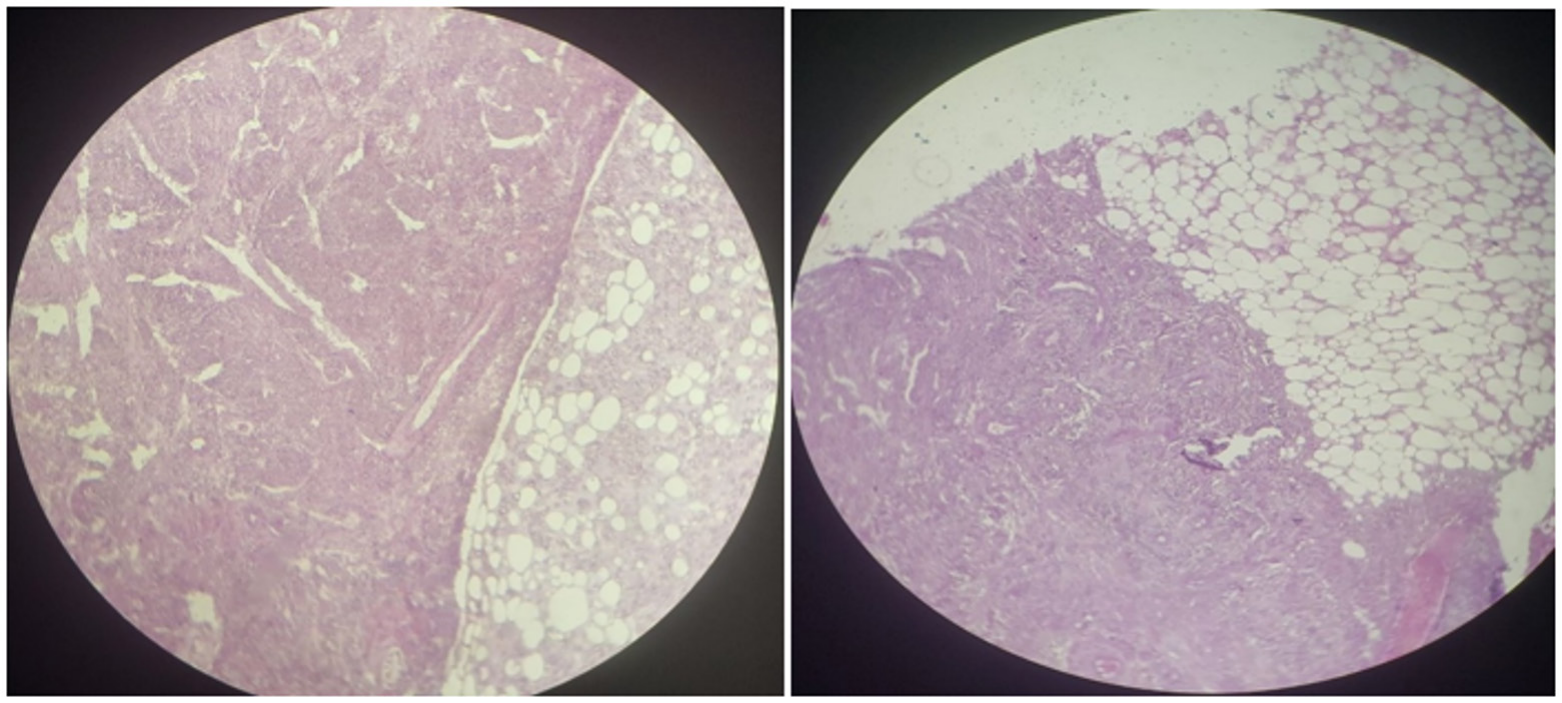
The tissue fragment consists of mature adipose tissue, thick‐walled blood vessels, and myeloid cells with eosinophilic cytoplasm, characterized by stromal hyalinization confirming renal angiomyolipoma.

Because of worsening flank pain during follow‐up, surgical excision of a renal mass was undertaken. Histopathologic examination of the excised specimen demonstrated mature adipose tissue, thick‐walled blood vessels, and myoid cells with eosinophilic cytoplasm and stromal hyalinization, confirming renal angiomyolipoma (Figures 6 and 7).

Taken together, the presence of facial angiofibromas, hypomelanotic macules, multiple renal angiomyolipomas, pulmonary lymphangioleiomyomatosis, cortical tubers, and a subependymal nodule fulfilled the 2021 International Tuberous Sclerosis Complex Consensus Diagnostic Criteria for a definite diagnosis of TSC.

### 2.3 Treatment

Initial management focused on stabilization and symptomatic control of abnormal uterine bleeding. Oral iron supplementation was initiated to treat iron‐deficiency anemia, while combined oral contraceptive therapy was prescribed for menstrual regulation and reduction of bleeding. Partial improvement in symptoms was achieved. The patient's longstanding seizure disorder remained well controlled with phenobarbitone 100 mg orally once daily, which was continued throughout treatment and follow‐up.

Following identification of multisystem manifestations of tuberous sclerosis complex, a multidisciplinary management strategy was implemented. Pulmonary follow‐up was arranged for surveillance of lymphangioleiomyomatosis, including clinical monitoring and pulmonary function assessment when available. The patient also received counseling regarding respiratory symptoms and potential pulmonary complications. Because of progressive bilateral flank pain and the presence of large renal angiomyolipomas, she was referred to the urology service for further management. A partial nephrectomy was subsequently performed, resulting in successful excision of an 8 × 7 × 5 cm renal angiomyolipoma from the left kidney.

Following confirmation of the diagnosis, the patient was enrolled in coordinated multidisciplinary follow‐up involving neurology, pulmonology, nephrology/urology, gynecology, and dermatology services, with ongoing surveillance for organ‐specific complications associated with TSC.

### 2.4 Outcome and Follow‐Up

The patient was diagnosed with definite tuberous sclerosis complex (TSC) based on the presence of multiple major clinical and radiologic features, including facial angiofibromas, hypomelanotic macules, bilateral renal angiomyolipomas, pulmonary lymphangioleiomyomatosis (LAM), cortical tubers, and a subependymal nodule. Additional gynecologic evaluation demonstrated uterine myometrial lesions compatible with perivascular epithelioid cell tumors (PEComas), which were considered the most likely cause of her abnormal uterine bleeding.

Following initiation of combined oral contraceptive therapy and iron supplementation, the patient experienced partial improvement in menstrual bleeding and symptoms related to anemia. Her longstanding seizure disorder remained well controlled with phenobarbitone, and no breakthrough seizures were reported during follow‐up. Because of progressive bilateral flank pain and the presence of large renal angiomyolipomas, she underwent partial nephrectomy with successful excision of a left renal mass measuring 8 × 7 × 5 cm. Histopathologic examination confirmed renal angiomyolipoma. The postoperative course was uneventful, and her flank pain improved following surgery.

After confirmation of the diagnosis, the patient was enrolled in multidisciplinary follow‐up involving neurology, pulmonology, nephrology/urology, gynecology, and dermatology services. Ongoing surveillance was planned to monitor disease progression and identify potential complications affecting the renal, pulmonary, neurologic, dermatologic, and gynecologic systems. At the most recent follow‐up, the patient remained clinically stable and continued regular outpatient follow‐up.

## 3 Discussion

Tuberous sclerosis complex (TSC) is a multisystem genetic disorder characterized by the development of hamartomatous lesions involving the brain, skin, kidneys, lungs, heart, and other organs [1, 2]. Although the condition is often diagnosed during childhood because of epilepsy, neurodevelopmental abnormalities, or characteristic cutaneous manifestations, some patients remain undiagnosed until adulthood when a single organ manifestation dominates the clinical picture [5, 14, 15]. This case illustrates the diagnostic challenge of adult‐onset recognition of TSC, as the patient presented primarily with abnormal uterine bleeding (AUB), while the underlying multisystem disorder remained unrecognized despite a longstanding seizure disorder and characteristic dermatologic findings.

The diagnosis of TSC in this patient was delayed not because clinical clues were absent, but because manifestations evolved gradually and were initially evaluated in isolation. Her seizure disorder had been present since adolescence and was controlled with antiepileptic therapy, while facial angiofibromas and hypomelanotic macules had not previously prompted investigation for a neurocutaneous syndrome. Similar diagnostic delays have been described in adults with TSC, particularly in resource‐limited settings where access to advanced imaging, genetic testing, and subspecialty care may be limited [4, 10, 11, 12, 13].

This case emphasizes the importance of careful physical examination and recognition of characteristic dermatologic findings, which often provide important diagnostic clues even when genetic testing is unavailable [5].

An important feature of this case is the unusual gynecologic presentation. Abnormal uterine bleeding is among the most common reasons for gynecologic consultation; however, TSC is rarely considered during the routine evaluation of AUB. Reported gynecologic manifestations of TSC include uterine hamartomas, perivascular epithelioid cell tumors (PEComas), ovarian cysts, and menstrual abnormalities [5, 16, 17, 18]. Because these lesions may mimic more common conditions such as leiomyomas or adenomyosis, diagnosis can be delayed. In the present case, pelvic MRI demonstrated uterine myometrial lesions compatible with PEComas, and the persistent abnormal uterine bleeding served as the sentinel manifestation that ultimately led to identification of the underlying syndrome. This observation supports previous reports suggesting that unexplained gynecologic pathology should prompt clinicians to consider systemic disorders when additional clinical features are present [5, 16, 17, 18].

The diagnosis became apparent only after a comprehensive evaluation revealed characteristic multisystem involvement. Renal angiomyolipomas are among the most frequent extracranial manifestations of TSC and represent a major diagnostic feature [2, 14, 15, 19]. Similarly, pulmonary lymphangioleiomyomatosis (LAM), particularly in women, is a well‐recognized manifestation associated with significant morbidity [5]. Neuroimaging demonstrated cortical tubers and a subependymal nodule, both of which are major features included in the 2021 International TSC Consensus Diagnostic Criteria [5]. Collectively, the coexistence of neurologic, dermatologic, renal, pulmonary, and gynecologic manifestations fulfilled the criteria for a definite diagnosis of TSC despite the absence of molecular confirmation.

Management of TSC extends beyond treatment of the presenting complaint and requires lifelong surveillance for organ‐specific complications [5]. In this patient, management included treatment of iron‐deficiency anemia and abnormal uterine bleeding, continuation of antiepileptic therapy, surgical management of symptomatic renal angiomyolipoma, and enrollment in multidisciplinary follow‐up. Such an approach is particularly important because disease progression may occur in organs that are initially asymptomatic, including the kidneys, lungs, and central nervous system [5, 20]. Early recognition therefore allows implementation of surveillance strategies that may reduce morbidity and improve long‐term outcomes.

In conclusion, this case highlights abnormal uterine bleeding as an uncommon sentinel manifestation of tuberous sclerosis complex. The diagnosis was established only after recognition of characteristic multisystem involvement, including dermatologic, neurologic, renal, pulmonary, and gynecologic features. Clinicians should maintain a high index of suspicion for syndromic disorders when evaluating atypical gynecologic presentations, particularly when accompanied by findings involving other organ systems. Early diagnosis facilitates multidisciplinary management and appropriate long‐term surveillance, which remain central to improving outcomes in patients with TSC.

## Author Contributions

**Kidus Tesfaye Bezabih:** conceptualization, investigation, funding acquisition, writing – original draft, validation, writing – review and editing, formal analysis, supervision, resources. **Hana Sisay Belay:** supervision, resources, data curation, formal analysis, software, validation, writing – review and editing, writing – original draft. **Merid Lemma Kebede:** conceptualization, investigation, validation, methodology, formal analysis, supervision. **Mathyas Dessalegn Gebreyes:** conceptualization, investigation, methodology, software, supervision. **Kedir Negesso Tukeni:** conceptualization, investigation, funding acquisition, writing – original draft, methodology, visualization, validation, writing – review and editing, software, formal analysis, project administration, data curation, supervision, resources. **Elias Fikre Merid:** conceptualization, investigation, methodology, validation, formal analysis, supervision, data curation. **Kelil Hussein Jibicho:** conceptualization, funding acquisition, visualization, validation, methodology, software, formal analysis, supervision. **Adugna Assefa Talemahu:** conceptualization, writing – original draft, writing – review and editing, project administration, data curation. **Abebe Ketema Aderu:** supervision, project administration, formal analysis, methodology, visualization, writing – review and editing, investigation.

## Funding

The authors have nothing to report.

## Ethics Statement

Ethical approval was unnecessary, but participants' information was kept confidential and used exclusively for this publication without identifying patients.

## Consent

Written informed consent was obtained from the patient for the publication of this case report and images, available for review by the Editor‐in‐Chief.

## Conflicts of Interest

The authors declare no conflicts of interest.

## Data Availability

The data that support the findings of this study are available from the corresponding author upon reasonable request.
